# Hierarchical Classification of Protein Folds Using a Novel Ensemble Classifier

**DOI:** 10.1371/journal.pone.0056499

**Published:** 2013-02-20

**Authors:** Chen Lin, Ying Zou, Ji Qin, Xiangrong Liu, Yi Jiang, Caihuan Ke, Quan Zou

**Affiliations:** 1 School of Information Science and Technology, Xiamen University, Xiamen, China; 2 College of Oceanography and Environmental Science, Xiamen University, Xiamen, China; 3 Shenzhen Research Institute of Xiamen University, Shenzhen, China; University of Westminster, United Kingdom

## Abstract

The analysis of biological information from protein sequences is important for the study of cellular functions and interactions, and protein fold recognition plays a key role in the prediction of protein structures. Unfortunately, the prediction of protein fold patterns is challenging due to the existence of compound protein structures. Here, we processed the latest release of the Structural Classification of Proteins (SCOP, version 1.75) database and exploited novel techniques to impressively increase the accuracy of protein fold classification. The techniques proposed in this paper include ensemble classifying and a hierarchical framework, in the first layer of which similar or redundant sequences were deleted in two manners; a set of base classifiers, fused by various selection strategies, divides the input into seven classes; in the second layer of which, an analogous ensemble method is adopted to predict all protein folds. To our knowledge, it is the first time all protein folds can be intelligently detected hierarchically. Compared with prior studies, our experimental results demonstrated the efficiency and effectiveness of our proposed method, which achieved a success rate of 74.21%, which is much higher than results obtained with previous methods (ranging from 45.6% to 70.5%). When applied to the second layer of classification, the prediction accuracy was in the range between 23.13% and 46.05%. This value, which may not be remarkably high, is scientifically admirable and encouraging as compared to the relatively low counts of proteins from most fold recognition programs. The web server Hierarchical Protein Fold Prediction (HPFP) is available at http://datamining.xmu.edu.cn/software/hpfp.

## Introduction

Information on proteins is crucial for understanding cellular organization and function [1], [2]. For each new protein sequence, sequence-sequence and sequence-structure comparisons are used to predict its possible function, but only the latter method of comparison remains accurate in identifying structurally similar proteins that lack sequence similarity [3]. The analysis of three-dimensional (3D) protein structures is one of the more efficient tools in molecular biology, cell biology, biomedicine and drug design [4]. However, the local minimum problem makes prediction of the overall protein folding difficult even when the direct prediction of the 3D protein structure is reliable [5]. The lack of proteins of known structure in datasets that are homologous to the query protein is an obstacle even when the homology modeling approach [6], [7] successfully predicts the 3D structure of a protein. Fold pattern prediction, which represents a deeper level of analysis than protein structural classification [4], lies between trapped secondary structure prediction and the partially effective tertiary structure prediction. Fold patterns are directly related to protein functions, and their prediction is critical, since these patterns can efficiently enhance the success rate of protein fold classification. Previous studies have indicated that protein fold recognition is urgently required in drug production [8], cancer therapy [9], and human immunodeficiency virus (HIV) treatment [10].

Proteins are considered to have a common fold pattern if they have the same major secondary structures with the same arrangement and topology [11]. Fold recognition refers to the recognition of the structural fold of a protein based on the given sequence information [12], and the number of possible protein folds is assumed to be restricted [13]–[16]. Therefore, prediction depends on the context of particular 3D folds. The decreased rate at which structural data containing new folds are entered into the *Protein Data Bank* (PDB), and the slowing addition of related Structural Classification of Proteins (SCOP) categories, indicates that the entire protein structural space will soon be fully covered. The large scale of the data makes fold prediction for a query sequence difficult. Several ensemble classification approaches have been presented to address this problem, including feature extraction and introducing more ensemble principles. Previous studies are mostly based on the class label of each protein sequence, or focus only on the 27 major folds [3], [4], [11]. Major disadvantage of such methods is that the 27 folds are represented in seven or more proteins and account for all major structural classes, hence it is insufficient for protein folds recognition. By investigating *support vector machines* (SVMs) and *neural networks* (NNs) (which can efficiently predict types of alpha-turns [17]), their study achieved an accuracy of 45.6% [3]. Since then, several ensemble classifiers have been utilized to reach a higher success rate. Two ensemble methods, *Discretized Interpretable Multi Layer Perceptrons* (DIMLPs) [18] and *Specialized Ensemble* (SE) [11], were developed using the stringent benchmark dataset, and the success rate of these methods reached 46.7% and 53%, respectively. Shen and Chou [19] later established an ensemble predictor called PFP-Pred, based on protein fold prediction, to achieve 62.1% accuracy with the same dataset. Another novel classifying method, PFRES, proposed by Chen and Kurgan [20], used a smaller number of more effective features and attained an accuracy of 68.4%. The PFP-FunDSeqE predictor was subsequently used with chained functional domains and sequential evolution information to achieve a success rate of 70.5% [4], which surpassed other ensemble classifiers. Chen [21], in a more recent paper, achieved 77% accuracy using an effective feature extraction method and a novel ensemble classifier. All the aforementioned experiments were developed using the benchmark dataset that was promoted by Ding and Dubchak [3], which is not satisfactory for the classification of all specific protein folds. Moreover, the current success rate requires further improvement.

In this study, we focused on improving the effect of protein fold pattern prediction. Using the latest SCOP release (version 1.75) [22], we deleted similar protein domains and reduced the homology of dataset to train a highly reliable model. For feature extraction, we considered the composition, distribution and physicochemical properties of amino acids (AAs) to obtain 188-dimensional (188D) features. The *Random Forest* (RF) model [23] was first adopted as a benchmark for our ensemble classifier. A particular training set may have several peculiarities, and merging numerous base classifiers can potentially reduce the deviation. Based on the processed dataset, a novel ensemble classifier was used to further enhance classification accuracy. We employed 18 base classifiers and several selective strategies to assemble highly variable classifiers to compensate for their individual disadvantages. Consequently, the classification results of seven protein classes were improved. Therefore, for the first time a hierarchical classification framework was developed in which all protein folds were recognized. The outcome of protein fold recognition was improved by the abovementioned methods with respect to: 1) improved classification accuracy using the novel ensemble classifier; and 2) the expansion of the prediction scope for protein folds using the hierarchical classification framework.

## Methods

Several procedures were developed to address the issues of protein fold recognition. These procedures included the extraction of feature vectors to establish the model, the integration of base classifiers to improve accuracy, and the prediction of the second layer of the SCOP database to handle the overall protein folds.

### Feature Extraction

In some cases, two proteins may be structurally similar but have no significant sequence similarity. More rational predictions are based on structural information, which are extracted as feature vectors according to the composition, distribution and physicochemical properties of the AAs in a specific protein [24]. An intermediate step that converts the sequence into a feature space representation should be performed, which could dramatically affect the prediction results.

Inspired by the work of Cai et al. [25], our present method considered AA composition as well as the content, distribution, and bivalent frequency of AAs possessing a variety of physicochemical properties [21] (listed in Table 1). First, the respective quantities of the 20 AAs (which are represented as AA_1_, AA_2_, … , AA_20_, alphabetically) were calculated as *n*_1_, *n*_2_, … , *n*_20_. . Accordingly, the feature vector (*FV*) (1–20) was denoted as:
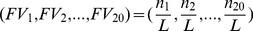
(1)where *L* is the sequence length.

**Table 1:** Division of amino acids into 3 different groups by different physicochemical properties.

physicochemical property	the 1st class	the 2nd class	the 3rd class
hydrophobicity	RKEDQN	GASTPHY	CVLIMFW
normalized Van der Waals volume	GASCTPD	NVEQIL	MHKFRYW
polarity	LIFWCMVY	PATGS	HQRKNED
polarizability	GASDT	CPNVEQIL	KMHFRYW
charge	KR	ANCQGHILMFPSTWYV	DE
surface tension	GQDNAHR	KTSEC	ILMFPWYV
secondary structure	EALMQKRH	VIYCWFT	GNPSD
solvent accessibility	ALFCGIVW	RKQEND	MPSTHY

**Figure 1:**
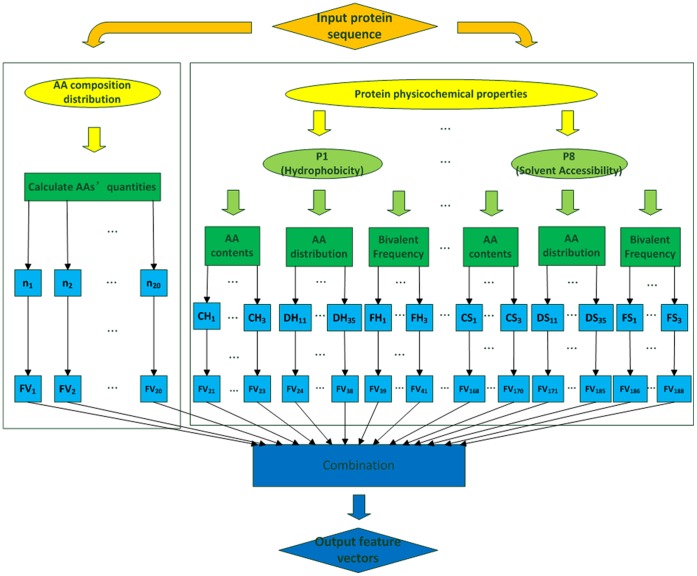
Extraction process of the 188-dimensional (188D) feature vectors (FV). Sequences are input and processed by analyzing amino acid composition, distribution and protein physicochemical properties, FV1–FV188 are output as feature vectors.

Next, we divided the AAs into three groups for each physicochemical property. Three descriptors, namely, the content (*C*), distribution (*D*), and the bivalent frequency (*F*), were used to describe the properties of each protein. Taking hydrophobicity (*H*) as an example:

1. The AAs were distributed to the RKEDQN, GASTPHY, and CVLIMFW groups according to their *H* properties. Using the size of the three groups (*CH*_1_, *CH*_2_, and *CH*_3_), we calculated *FV* (21–23) as:

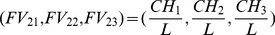
(2)2) The chain length was measured as *DH_ij_* (*i* = 1, 2, 3; *j* = 1, 2, … , 5), wherein the first, 25, 50, 75, and 100% of AAs of a particular property were located, respectively. Then, we defined *FV* (24–38) as:

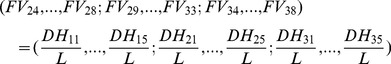
(3)3) The number of bivalent seeds was represented (*L* – 1), and we counted the respective number of bivalent seeds that contained two AAs from different groups. Then, we obtained the parameters *FH*_1_, *FH*_2_, and *FH*_3_ to define:

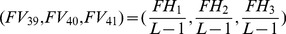
(4)A total of 21 feature vectors were calculated for each property. After all physicochemical properties were analyzed, we finally extracted all 188 feature vectors. A flowchart to show this specific process is presented in Figure 1.

### Ensemble Classifier

To achieve satisfactory results, our ensemble classifier combines different base classifiers to significantly improve accuracy.

**Figure 2:**
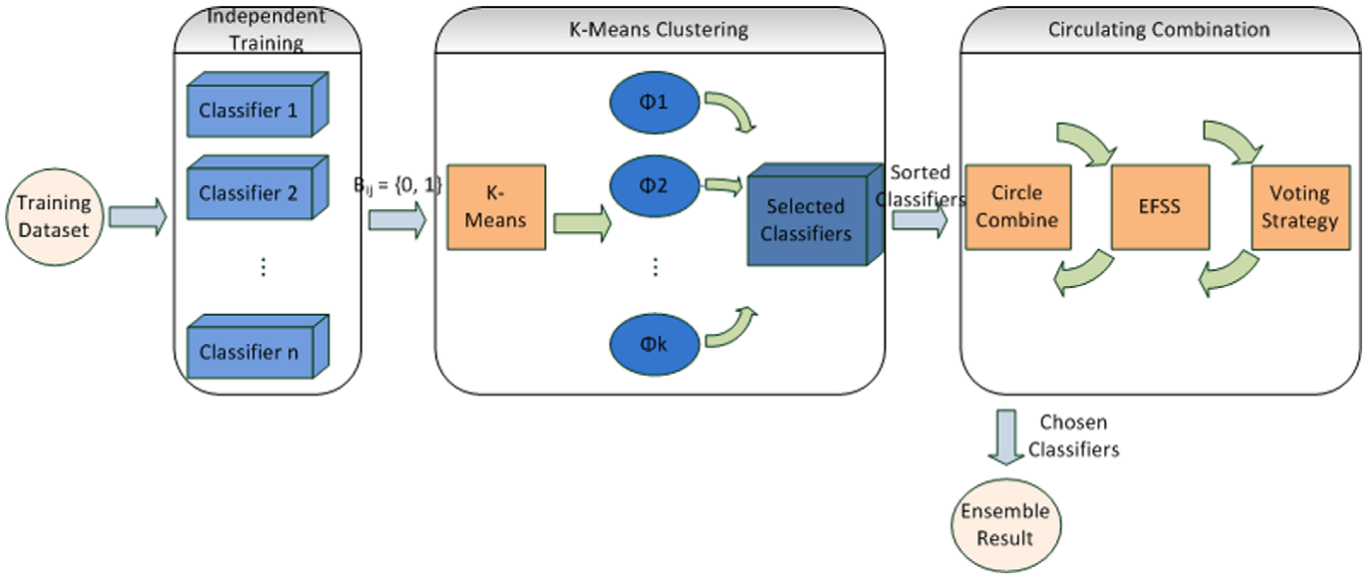
The architecture of our ensemble classifier. The training dataset is classified by all base classifiers. After K-Means clustering and circulating combination the best ensemble result is achieved.

Knowing that appropriately combined ensemble classifiers can optimize the prediction effect [26], we attempted to find an effective ensemble practice. Previous research indicated that the diversity of the base classifiers facilitates further improvement. Accordingly, we utilized a K-Means clustering algorithm [27] to choose a series of discrepant base classifiers and a circulating, combined static selective strategy, *Ensemble Forward Sequential Selection* (EFSS). EFSS employs the vote rule for its ensemble. In this way, the proper classifiers were ultimately acquired. Figure 2 illustrates the architecture of our ensemble classifier.

In order to further improve the classifier, we considered the problem of classifying a given dataset through an ensemble of *n* = 18 basic classifiers, which were designated as *C*1, *C*2, …, *C*18. The specific classifier algorithms that were used in this study are: 1) Logistic Regression, 2) SMO^1^, 3) SVM^2^, 4) IB1^3^, 5) IB5, 6) IB10, 7) OneR^4^, 8) Conjunctive Rule, 9) Decision Table, 10) JRip^5^, 11) ZeroR^6^, 12) Simple Cart, 13) Naïve Bayes, 14) Random Tree, 15) FT Tree^7^, 16) RF^8^, 17) Decision Stump, and 18) J48^9^. The base classifiers train the primitive entities independently. The results were represented as *B_ij_* = {0, 1} (*i* = 1, 2, …,18; *j* = 1, 2, …, *m*), where *m* is the number of instances. *B_ij_* = 0 indicates that classifier *i* failed to predict instance *j* and vice versa. The resulting matrix flows to K-Means clustering, as shown in Figure 2.

We set the partition number to *k* = 9, such that the K-Means algorithm divides the base classifiers into nine clusters. Details of K-Means technique are described in [27]. The classifier with the best performance in each cluster was chosen to generate a set of selected classifiers.

To further improve the method, a circulating combination methodology was employed, after classifiers from the output set are sorted in descending order by their classification accuracy. We created another chosen classifiers (CC) set to record the selected classifiers. In each circle, EFSS successively chooses the best performing classifiers and creates an ensemble with the classifiers in CC according to the vote rule. If its diversity decreased as well as its accuracy increased, the chosen classifier was added to the group of CCs. Circulation continued until the final result surpassed our target accuracy, which is reduced by one step in each circle. The definite algorithm 1 is presented in Table 2. Before using the algorithm, we initialized the target accuracy (TA) to 1, the optimal accuracy (OA) to 0, the step to 0.05, and the current result, which contains three parameters. The diversity is set to be a very large number to represent infinity, whereas the success rate and the chosen number are zero.

**Table 2:** Algorithm 1. Circulating Combination of EFSS.

Input: Sorted Classifiers set SC and Training Dataset T
Output: Chosen Classifiers set CC
while TA > = 0 and OA <TA
while SC is not empty and success rate < TA
choose the first element C0 in SC
ensemble CC U C0 by voting strategy and train T
if diversity decreases and success rate increases
CC.append(C0)
end
remove C0 from SC
end
if success rate >OA
OA : = success rate
end
if OA < TA
TA : = TA – step
end
set SC with initial data
end

The ensemble classification problem was successfully resolved in this study for the first time and K-Means clustering was used to select the most diverse classifiers. The voting strategy of circle combination used in the EFSS allowed us to achieve the best combination of classifiers. Consequently, our novel ensemble classifier, which multiplies selection strategies, is superior to those which simply choose several highest-rated classifiers that are then immediately used for the ensemble.

### Hierarchical Classification Framework

**Figure 3:**
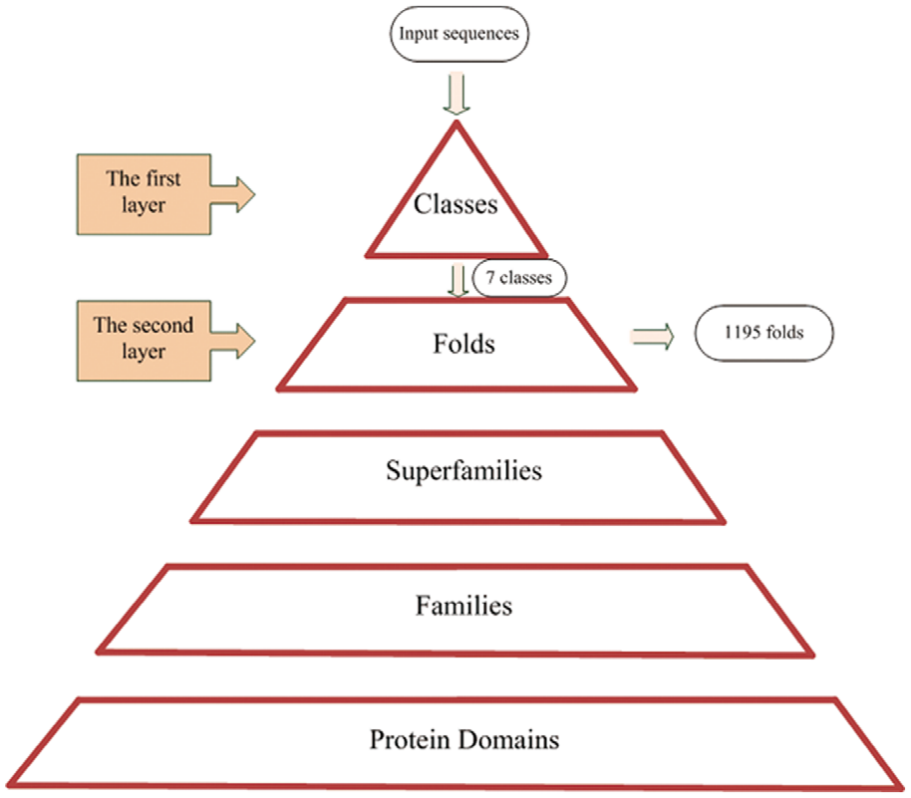
Protein structure levels in SCOP. The classification of protein classes and of protein folds are the first and second layer, respectively, of our hierarchical classification frame.

SCOP (version 1.75) was used as the data source in our experiment. This dataset classifies protein structures hierarchically based on evolutionary relationships and on the principles that govern their 3D structure [16]. The levels of protein structure are displayed in Figure 3, and the protein domain is the unit of classification. If all proteins in a group have residue identities of 30% and greater, or if proteins with lower sequence identities are similar in function and structure, this group of proteins can be denoted as a family. Families with proteins of low sequence identities that have structures and functions suggestive of a common evolutionary origin are grouped together in a superfamily. When proteins have the same major secondary structures similar in arrangement and topological connections, these proteins are classified as part of a common fold, which is the ultimate target of our classification process. For further convenience, the different folds are grouped into seven classes: the all-α proteins (284 folds), all-β proteins (174 folds), α/β proteins (147 folds), α + β proteins (376 folds), multi-domain proteins (66 folds), membrane and cell surface proteins and peptides (58 folds), and small proteins (90 folds). These classes comprise the first layer of our hierarchical classification framework.

Since the structure of the SCOP (version 1.75) database is hierarchical, the database can readily be used to verify a hierarchical framework. As shown in Figure 3, we first import the dataset into the first layer. After RF or ensemble classification, we obtained high-accuracy results for the seven classes. For each class, the protein sequences were trained in the second layer and classified into 1195 folds with lower accuracy. The framework and the prediction model have been structured. Consequently, upon the arrival of a new sequence, the sequence is tested in the hierarchical framework in succession to eventually be predicted as a fold.

Previous studies [3], [4], [11], [19]–[21] have classified protein structures into four classes or 27 folds, which are each present in at least seven proteins and which represent all major structural classes. Such an approach applies only to a certain number of proteins. For proteins that belong to less populated folds, their effects on the recognition results are neglected. To overcome this limitation, we proposed a hierarchical classification framework. For the first time, all protein folds were considered in order to improve the precision of the predictions.

## Experiments

To improve the classification accuracy and to expand the prediction scope, we developed a series of experiments to validate the effectiveness and efficiency of our methods. In this section, we discuss the dataset that was used. Then, we describe our analysis of the experiment results using the previously-mentioned methods, and our testing of the effectiveness of the individual feature sets from the proposed sequence representation.

### Data

**Figure 4:**
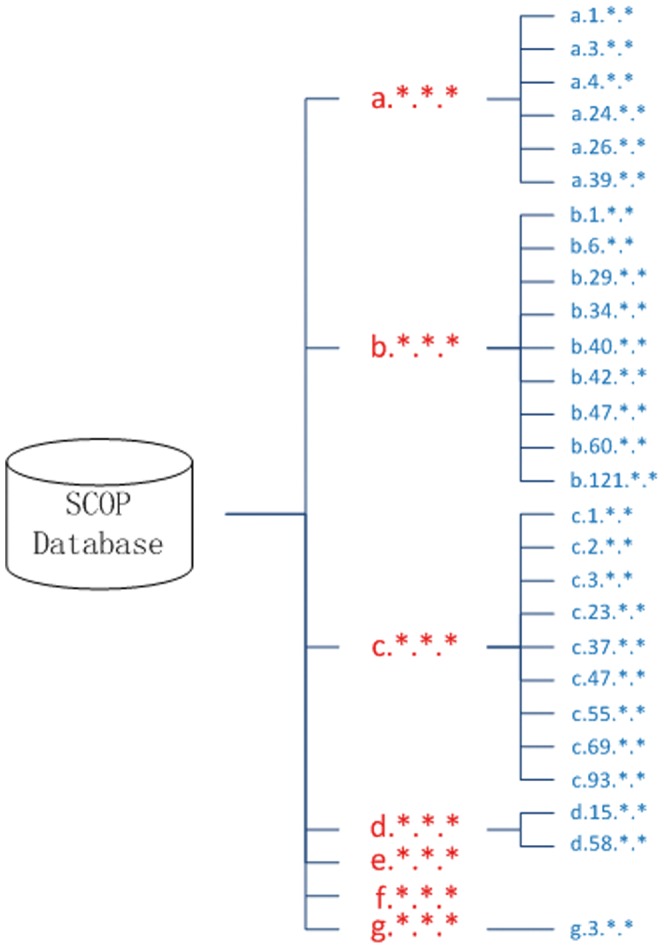
Comparison of the two datasets. In each query description, the first letter represents the class name and the second digit represents the fold number. The SCOP dataset that was used in this paper is shown in red. Seven classes containing 1195 folds (which are omitted in the figure) from SCOP dataset are explained as: (a) all-α proteins (284 folds), (b) all-β proteins (174 folds), (c) α/β proteins (147 folds), (d) α+β proteins (376 folds), (e) multi-domain proteins (66 folds), (f) membrane and cell surface proteins and peptides (58 folds), and (g) small proteins (90 folds). The benchmark dataset [3] proposed by Ding and Dubchak, composed of the 27 folds that were extracted from SCOP, is shown in blue.

SCOP is a database of protein structural classification which provides a detailed and comprehensive description of the structural and evolutionary relationships of proteins, including all entries in the *Protein Data Bank* (PDB). SCOP (version 1.75) was released in June, 2009 [22] and is used in the current work. While the dataset of Ding and Dubchak [4], which includes 27 of the 1195 protein folds, is widely used, this dataset is outdated. The stringent benchmark dataset is compared with the SCOP database in Figure 4. We anticipated that the latest version of the SCOP database would lead to more precise and credible predictions.

The latest release of the SCOP database contains a total of 105,725 protein sequences, which include the 17,051, 26,552, 28,304, 25,536, 2,192, 1,874, and 4,216 protein sequences that belong to classes (a) to (g), respectively. While handling this dataset, we discovered that it contains a high level of redundancy. For example, several protein pairs were identical or very similar in sequence. Statistical analyses of proteins require non-homogeneous data because the set of selected structures should be a sufficient representative of the whole [28]. Based on sequence identity [29], [30], the general measurement of protein sequence similarity, we compared prediction effects of sequence subsets at various levels of redundancy and obtained datasets in a series of frequently used identity values. The most representative dataset possesses upon 35% sequence identity, which is the same as the one used by Ding and Dubchak [3]. Along with the decrease in identity, the sequences have become more independent.

Another measurement we used to extract non-homogeneous data was the edit distance, in which we selected longer sequences in pairwise alignments performed by a partition-based method pass-join [31] with different edit distances. The selection process and predicting performance are referred to in Appendix S1.

### Classification Performance

**Figure 5:**
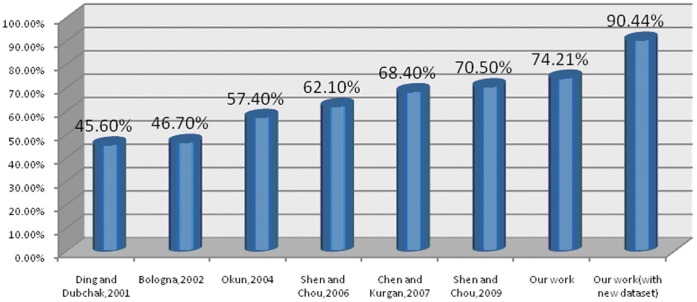
Comparison of success rate among several studies. Our work outperforms all previous works with an accuracy of 74.21%.

Through a set of experiments, we achieved highly satisfactory classification performance. In this subsection, we present experimental data that demonstrate the enhanced classification accuracy - the rate of measurement results to success - of the present method. Figure 5 compares our success rate to that of previous studies.

We utilized two datasets to validate our comparison. Dataset 1 is the same dataset that was used in the first six studies. We combined the training and testing sets in the benchmark dataset, determined the different features, developed the classification method with our ensemble classifier, and obtained an accuracy of 74.21%, which is the highest level of accuracy that has been recorded. Dataset 2 is extracted directly from the SCOP database and contains the same 27 folds found in the first dataset. Using the new dataset increased accuracy to 90.44%. We attribute this higher accuracy to two reasons: the updated SCOP facilitates the precision of classification, and the redundancy of the data increases the biased success rate.

**Figure 6:**
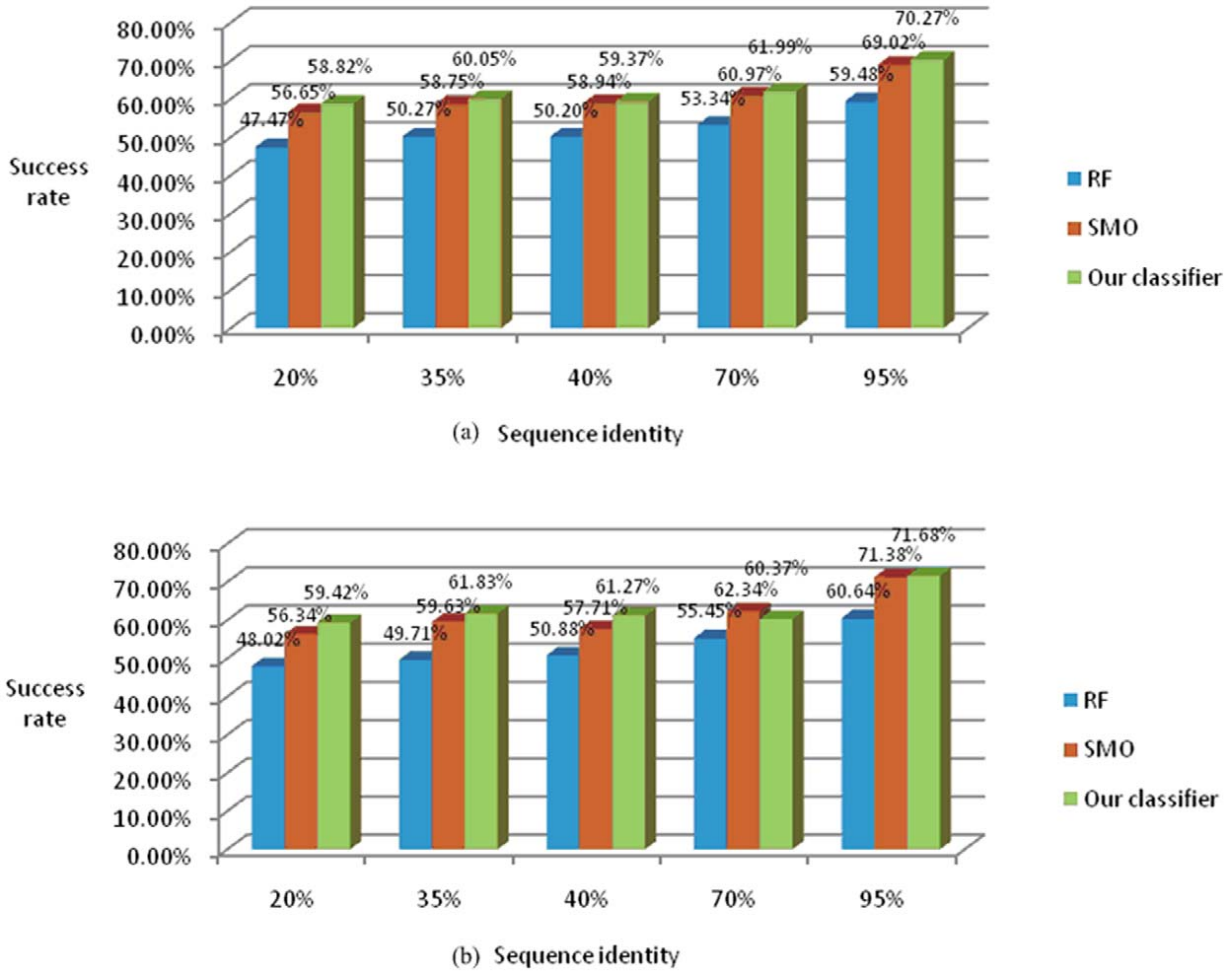
Success rate achieved by three classifiers with different sequence identity. The two graphs show the results of two datasets((a) SCOP version 1.75, (b) SCOP version 1.75A). Their similar success rates demonstrate the robustness of our model. As identity increases it becomes less stringent and success rate rises. It also shows our ensemble classifier outperforms other two classifiers.

Since the effectiveness of our methods has been proven, we focused on the use of the latest processed dataset, which was previously described in Section 3.1. We also utilized the newest transitional version (version 1.75A) of SCOP to verify the universal applicability of our method. We classified several datasets into seven classes using tenfold cross-validation, as shown in Figure 6. The change of classification accuracy among different datasets is illustrated in Figure 6.

The first layer of the histogram shows an increasing trend of accuracy as sequence identity becomes less stringent. The success rate lies between 50.27% and 60.05% at the identity of 35%. Although this success rate is outperformed by previous researches, considering the existence of decentralized and less related data in each class, which contains hundreds of folds, the results are actually satisfactory. In the second layer, datasets from SCOP version 1.75A also showed a high accuracy. Our model can therefore be applied to new datasets rather than being confined to our own experimental data. Figure 6 shows that, our novel ensemble classifier significantly outperformed the other two classifiers. To further analyze this disparity, we chose several promising base classifiers according to the output of ensemble classifiers (Table 3 and Table 4).

**Table 3:** Performance on different classifiers on protein fold recognition (one sequence in each family).

Classes	Random Forest	SMO	Logistic	Ib1	Ib10	Naïve Bayes	Decision Table	Our classifier
all-α proteins	54.1%	63.9%	52.8%	38.3%	46%	17.9%	54.6%	61.7%
all-β proteins	42.8%	54.4%	38.4%	31.4%	32.5%	17.4%	41%	58.9%
α/β proteins	57.1%	61.7%	58.7%	46%	59.9%	35%	55.5%	66%
α+β proteins	37.7%	45.9%	41%	36.4%	37.2%	31%	45.7%	49.3%
multi-domain proteins	5.7%	0	23.9%	13.6%	1.1%	76.1%	1.1%	80.5%
membrane and cell surface proteins and peptides	36.9%	53.3%	54.7%	38.5%	32.8%	8.2%	7.4%	28.6%
small proteins	67.4%	82.6%	6.8%	56.9%	56.9%	83%	39.9%	88.7%
Total accuracy	47.3%	56%	44.9%	38.5%	43.1%	29.5%	46.3%	59.6%

**Table 4:** Performance on different classifiers on protein fold recognition (sequence at 35% identity).

Classes	Random Forest	SMO	Logistic	Ib1	Ib10	Naïve Bayes	Decision Table	Our classifier
all-α proteins	53.5%	62.1%	52.8%	39%	44.9%	16%	47.4%	64.3%
all-β proteins	49.5%	57.6%	43.6%	35.6%	40.3%	29.4%	42.7%	51%
α/β proteins	66.4%	73.1%	65%	52.5%	73.3%	38.8%	68.5%	69.6%
α+β proteins	31.3%	41.9%	40.6%	35.4%	31.6%	29.8%	35.2%	54.2%
multi-domain proteins	5%	0	28%	15.6%	6.7%	78.2%	0	58.3%
membrane and cell surfaceproteins and peptides	37.3%	50.3%	38.8%	39.4%	28%	12.4%	3.1%	41.9%
small proteins	75.1%	85.7%	65.4%	68.5%	68.8%	89.2%	59.5%	70.7%
Total accuracy	50.3%	58.7%	50.9%	42%	48%	33.3%	47.5%	60.1%

To demonstrate the robustness of the results, we processed the dataset in two ways. A protein family is defined as a group of proteins with residue identities of 30% and greater. We extracted the longest sequence of each family and obtained a new training set, in which tenfold cross-validation shows that over-fitting has been avoided.

The other dataset is the subset with an identity of 35%, as described in Section 3.1, which was used in our later experiment.

Table 3 shows that the accuracy using our novel ensemble classifier is 59.61%, which proved to be acceptable. Table 4 shows that, the all-β and the α/β classes are the easiest to classify. While the accuracy of the base classifiers ranges between 33.3% and 58.7%, the ensemble classifier achieves an accuracy of 60.1%. These results represent an effective combination of base classifiers and explain the improved protein fold prediction in our work.

### Feature Analysis

With the exception of the effect of the ensemble classifier, the feature extraction method contributed to the classification results in the previous section. Our method is based on the composition, distribution, and physicochemical properties of the AAs in a specific protein. To determine which variable has the most influence on the classification results or which feature vector most influences information in the dataset, we designed an experiment that uses the normative Principal Component Analysis (PCA) method.

PCA is a simple, non-parametric method for extracting relevant information from confusing datasets that has become a standard tool in modern data analysis. PCA is mathematically defined as an orthogonal linear transformation that transforms data to a new coordinate system such that the greatest variance by any projection of the data lies on the first coordinate (called the first principal component), the second greatest variance on the second coordinate, and so on. Therefore, the final features are the linear combination of the original features to guarantee that the principle component is independent and belongs to a lower dimension. By choosing proper components and analyzing the corresponding component loadings, the ranking of variables can be addressed.

The dataset performs best when the edit distance is set to 1, and this dataset was further utilized in our feature analysis experiment. The dataset was subjected to PCA analysis with the default parameters. The first column of Table 5 shows the different components that are ordered by eigenvalue, whereas the other three columns show the eigenvalues, percent contribution, and cumulative percent contribution for a portion of components extracted from the correlation matrix. Our feature extraction method considers amino acid composition and distribution as well as eight physicochemical properties, giving a total of nine factors, along with the first ten components that account for nearly 50% of the total variance. Given that their eigenvalues are apparently greater than those of other components, we decided to retain these first ten components for subsequent analysis.

**Table 5:** Preliminary results* of PCA analysis.

Component	Eigenvalue	Percent explained	Cumulative percent explained
1	20.00517	0.10641	0.10641
2	14.2717	0.07591	0.18232
3	12.47311	0.06635	0.24867
4	10.11524	0.0538	0.30247
5	8.69996	0.04628	0.34875
6	8.05621	0.04285	0.3916
7	6.18316	0.03289	0.42449
8	5.10462	0.02715	0.45164
9	4.77623	0.02541	0.47705
10	4.25826	0.02265	0.4997
11	4.01185	0.02134	0.52104
12	3.76333	0.02002	0.54106
13	3.43468	0.01827	0.55933
14	3.23814	0.01722	0.57655

* Eigenvalue > 3.

The optimal PCA result contains 88 components, from which the first ten were extracted to illustrate their loading (Table 6). According to our feature extraction method, the 188 features represent the AA composition and attributes of the respective eight physicochemical properties. Table 6 shows the uniformly distributed component loadings of the features, confirming that the factors we have chosen all contribute information that can discriminate between different proteins. Each component stands for a property. Consequently, the distribution of the three most contributive features is similarly even. The factors we have taken account, namely, the content, distribution, and the bivalent frequency, all supply necessary information for the classification model. Therefore, we conclude that the feature extraction method is effective.

**Table 6:** Loadings of most informative features* on principle component factors.

Feature	C1	C2	C3	C4	C5	C6	C7	C8	C9	C10
21	−0.186									
29		0.189								
34				−0.179v						
43			0.151							
44					−0.171					
50				−0.176						
53			−0.138							
60						0.199				
63						0.221				
65	−0.186									
70					−0.173					
81		0.192								
86					−0.171				0.151	
92				−0.175						
110								−0.165		
126							−0.241			
127									−0.177	
128						0.231				
131							0.212			
134									0.161	−0.204
138										0.2
146			0.158							
148							0.2			
157								0.157		
168										0.202
169	−0.186									
175								0.156		
181		0.189								

* Only the first three are shown.

### Hierarchical Classification Performance

The prediction of protein folds is significant for subsequent studies. However, our previous work in Section 3.2 was only able to classify the proteins into their classes. Although its performance is exceptional, the overall effect is merely acceptable. Hence, we proposed a hierarchical classification framework to make additional efforts to predict the protein folds.

**Figure 7:**
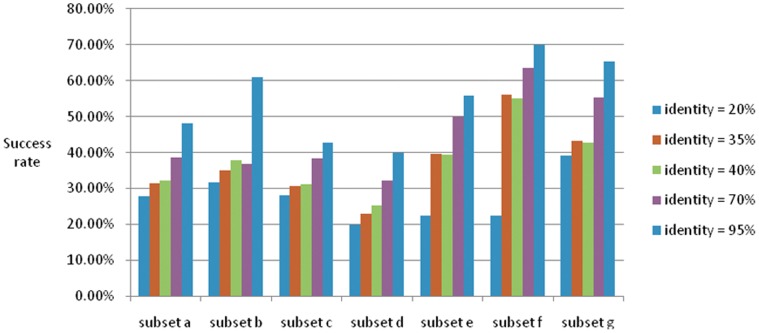
Success rate of seven subsets with different sequence identities. The figure shows factors influencing success rate. Success rate has an increasing trend when sequence identity rises or class number drops.

The entire dataset is split into seven subsets, each of which is processed with different sequence identities. We predicted each subset in the same way, and the general results are shown in Figure 7, which shows that the success rate increases as sequence identity is enhanced. When sequence identity equals 20%, the accuracies of seven subsets range between 19.89% (for subset d) to 39.22% (for subset g). When sequence identity approaches 95%, accuracies range between 39.92% (for subset d) to 70% (for subset f). Discrepancies are subtle among subsets but are significant when similar sequences are excluded.

The second level of the hierarchical frame displays much lower accuracy, especially when the sequence identity decreases. The second level performs better with massive training instances. . Specific data are listed in Table 7, from which we can determine the relationships between the class number, instance number and success rate. As the identity decreases, the instance number is reduced and the accuracy decreases accordingly, as shown in each row. By comparing the first and second layers of the framework, we conclude that the decreased accuracy is a result of the decreasing number of instances. While comparing the performance of the seven subsets, we can see the decreased accuracy results along with the rapid growth of the class number.

**Table 7:** Influential factors for success rate of 1^st^ and 2^nd^ hierarchical layers.

Sequence identity		20%	35%	40%	70%	95%
Subset a	Accuracy	27.81%	31.40%	32.16%	38.57%	48.07%
	Class number	285	285	285	285	285
	Instance number	1437	1883	1990	2464	2997
Subset b	Accuracy	31.65%	34.93%	37.73%	36.68%	61.04%
	Class number	175	175	175	175	175
	Instance number	1455	2026	2218	2966	4105
Subset c	Accuracy	28.00%	30.71%	31.02%	38.40%	42.76%
	Class number	148	148	148	148	148
	Instance number	1606	2434	2679	3529	3925
Subset d	Accuracy	19.89%	23.00%	25.19%	32.12%	39.92%
	Class number	377	377	377	377	377
	Instance number	1783	2480	2670	3389	3953
Subset e	Accuracy	22.30%	39.61%	39.32%	50.00%	55.79%
	Class number	67	67	67	67	67
	Instance number	122	179	191	240	273
Subset f	Accuracy	22.30%	56.11%	54.95%	63.65%	70.00%
	Class number	59	59	59	59	59
	Instance number	122	193	198	233	270
Subset g	Accuracy	39.22%	43.33%	42.75%	55.41%	65.45%
	Class number	91	91	91	91	91
	Instance number	438	558	626	916	1192

When the target class number *N* is in the range of hundreds, the ordinary prediction accuracy should be 1/*N*, that is, ∼1% of *N*. Therefore, our prediction accuracy (lowest = 19.89%) is sufficiently satisfactory. However, our prediction accuracy is not as high as that of the first layer. Furthermore, our study deals with the possible folds of all proteins. The performance of the proposed hierarchical framework will guide further work on protein fold recognition.

### Conclusions

Protein fold recognition has been an important aspect of bioinformatics research for several decades. In the present paper, we improve the fold pattern recognition results by enhancing prediction accuracy and expanding the forecast range.

In our preparatory work, we excluded redundant protein items in the latest SCOP database to build an unbiased prediction model. We extracted the feature vectors via the analysis of amino acid composition, distribution, and physicochemical properties. To enhance the success rate, we used a novel ensemble classifier, which circulates and combines the selected base classifiers based on clustering. To expand the classification range, we proposed a hierarchical framework. Using the second layer, all proteins could be classified into a fold. Accordingly, the overall classification effect becomes more precise and accurate.

Our experimental results proved to be effective and comprehensive. Using PCA analysis, we showed that feature extraction was possible. To demonstrate an improvement in the success rate, we first utilized the same dataset from previous studies to demonstrate an improved success rate using the current method. Our ensemble classifier performed with 74.21% accuracy, which outperforms the best result (70.5%) achieved by Shen and Chou [4] in 2009. In the present work, we classified the first and second layer of the hierarchical framework for the most recent dataset. For the first layer, performance was outstanding, with accuracy ranging between 58.83% and 70.27%. After further entering the data into the second layer, the success rate was much lower because of the increasing classification class number and the decreasing prediction instance number. Although the prediction result is satisfactory, the present results can still be improved. Future work will focus on increasing the accuracy of multi-classification with numerous classes.

In conclusion, our current work has evidently improved the prediction effect and will lead to other similar studies in this area.

## Supporting Information

Appendix S1 **Novel Measurement for Sequence Redundancy.** Different edit distances (0,1,3,5,10) were used for comparing the predicting precision. It showed the predicting influence of the redundance of the protein dataset.
(PDF)
