# Supplementary material for: Hierarchical Classification of Protein Folds Using a Novel Ensemble Classifier
Source: PLoS One. 2013 Feb 20;8(2):e56499. doi: 10.1371/journal.pone.0056499 (PMC3577917; doi:10.1371/journal.pone.0056499)
Supplement: Appendix S1 — Novel Measurement for Sequence Redundancy. Different edit distances (0,1,3,5,10) were used for comparing the predicting precision. It showed the predicting influence of the redundance of the protein dataset. (PDF) [file pone.0056499.s001.pdf]

Appendix S1.Novel Measurement for Sequence Redundancy.

In this paper, we propose a novel method to decrease protein sequence redundancy. Pairwise alignment of the sequences was performed using a partition-based methodpass-join [1] with a series of edit distances, which is defined as the minimum number of single-character edit operations (i.e., insertion, deletion, and substitution) needed to transform one sequence to another. Using a list of candidate proteins and their similarity relationships, we selected the longest sequence (with more structural information) in each round and judged whether the sequence is homogeneous with any sequence selected. If no similar sequences were found, the sequence was added into our selected set. After this process, we obtained a less redundant dataset in varying degrees, which are determined by edit distance. The changes in the sequence number of seven classes and in the whole dataset are shown in Figure S1. The primitive numbers are omitted in the figure because of the tremendous gap in their magnitude.

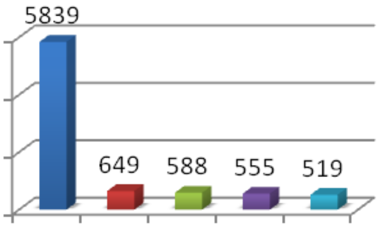

(a) class a

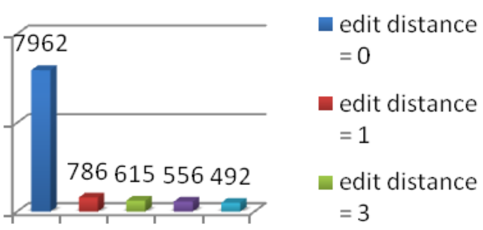

(b) class b

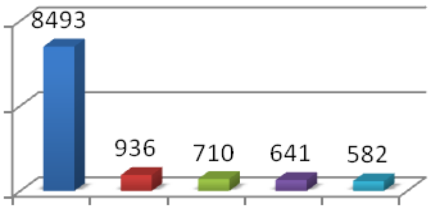

(c) class c

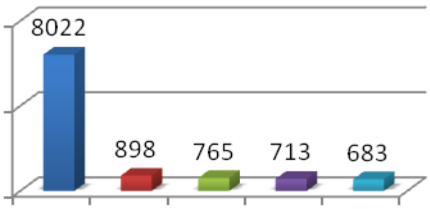

(d) class d

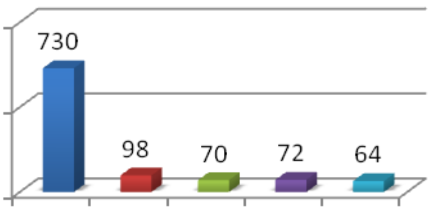

(e) class e

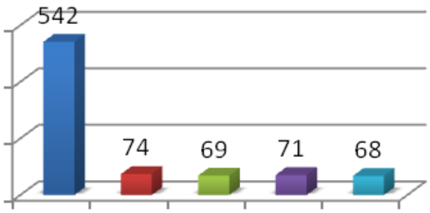

(f) class f

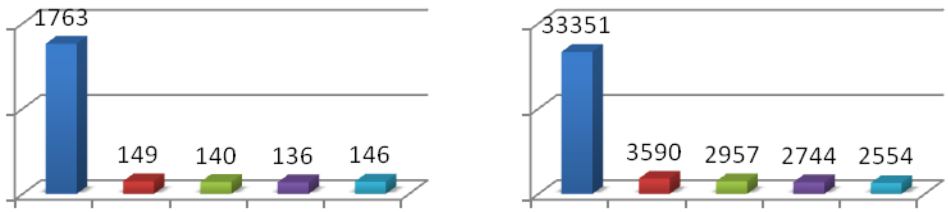

(g) class g (h) the whole dataset

Figure S1 **Changes in the number of sequences with edit distances of 0, 1, 3, 5, and 10.** An edit distance of zero means that exactly the same sequences were removed from the original database. Graphs represent the different protein classes in the dataset: (a) all-α proteins, (b) all-β proteins, (c) α/β proteins, (d) α+β proteins, (e) multi-domain proteins, (f) membrane and cell surface proteins and peptides, and (g) small proteins, as well as (h) the whole dataset.

Based on the definition of edit distance and the deletion result, we can conclude that the sequence identity, which is used in measuring the redundancy by Ding and Dubchak [2], becomes more stringent as edit distance increases. Sequences with larger edit distances are more different from one another. The dataset can then be utilized representatively in protein fold prediction and recognition. We calculated data at different edit distances using three classifiers. (Figure S2).

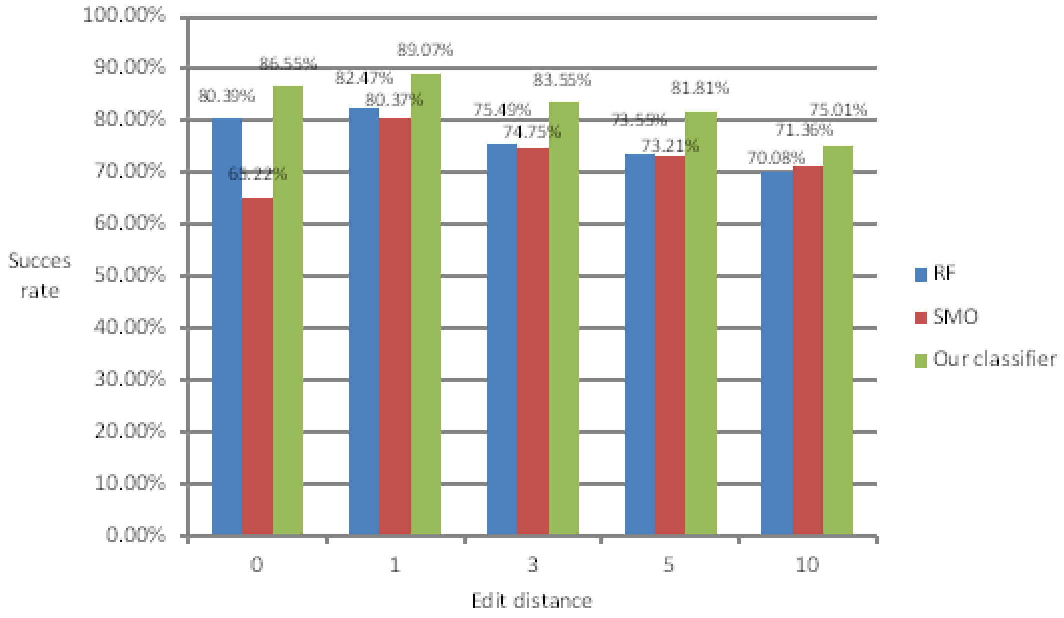

Figure S2 **Success rate achieved by three classifiers with different edit distances**

The first dataset excludes all protein sequences with that do not differ from one another. The edit distance between two protein sequences is*n*, if the two protein sequences are identical, except for *n* number of amino acids (regardless of type or position). Therefore, only one of the two identical sequences is retained.

Figure S2 shows that a decreasing trend is observed as edit distance increases. The accuracy is enhanced when the edit distance changes to 1 from 0 because of the more robust model, but decreases afterwards due to the smaller training set. Since the model behaves well with an edit distance of 1, the dataset was used further in our hierarchical classification framework (Figure S3).

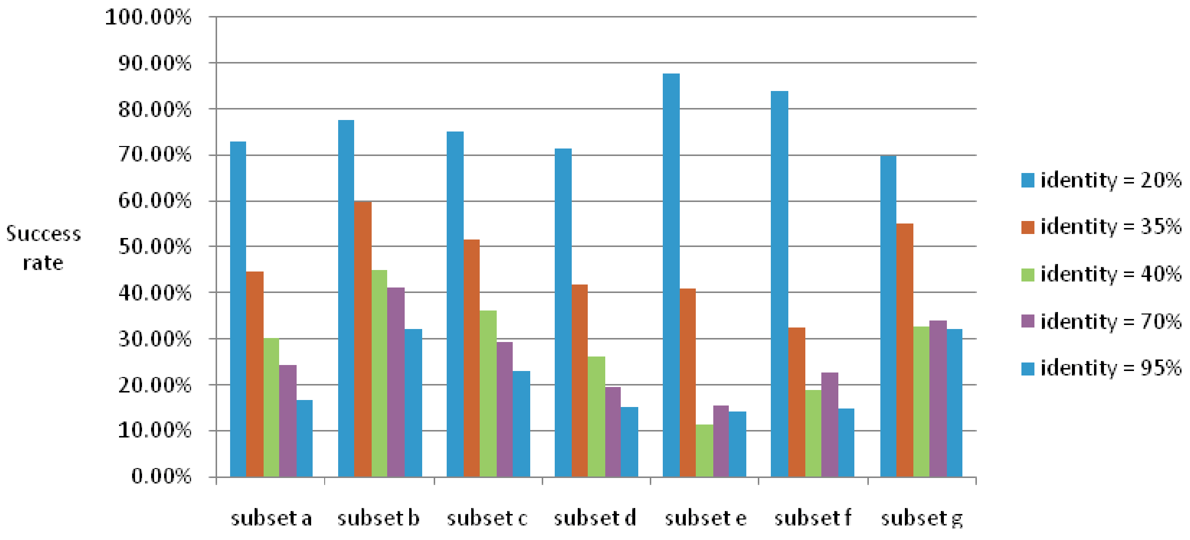

Figure S3 **Success rate of seven subsets with different edit distances**

Figure S3 also indicates a good performance in second layer of the classification. The descending trend as edit distance increases demonstrates our assumptions that edit distance is negatively correlated with sequence identity. However, they are both effective measurements of protein sequence redundancy.

Reference

[1] Jianhua Feng, Jiannan Wang, Guoliang Li. (2012)Trie-join: a trie-based method for efficient string similarity joins[J]. The VLDB Journal, 21(4):437-461.

[2] Ding CHQ and Dubchak I (2001). Multi-class protein fold recognition using support vector machines and neural networks[J]. Bioinformatics 17: 349-358.
